# Physical activity and lifestyle modifications in the treatment of neurodegenerative diseases

**DOI:** 10.3389/fnagi.2023.1185671

**Published:** 2023-05-26

**Authors:** Jose A. Santiago, Judith A. Potashkin

**Affiliations:** ^1^NeuroHub Analytics, LLC, Chicago, IL, United States; ^2^Center for Neurodegenerative Diseases and Therapeutics, Cellular and Molecular Pharmacology Department, The Chicago Medical School, Rosalind Franklin University of Medicine and Science, North Chicago, IL, United States

**Keywords:** neurodegeneration, neuroprotection, physical activity, dementia, Alzheimer's disease, Parkinson's disease, ALS

## Abstract

Neurodegenerative diseases have reached alarming numbers in the past decade. Unfortunately, clinical trials testing potential therapeutics have proven futile. In the absence of disease-modifying therapies, physical activity has emerged as the single most accessible lifestyle modification with the potential to fight off cognitive decline and neurodegeneration. In this review, we discuss findings from epidemiological, clinical, and molecular studies investigating the potential of lifestyle modifications in promoting brain health. We propose an evidence-based multidomain approach that includes physical activity, diet, cognitive training, and sleep hygiene to treat and prevent neurodegenerative diseases.

## Introduction

The World Health Organization (WHO) declared neurological disorders as the leading cause of disability and the second leading cause of death worldwide in 2022 (https://www.who.int/). The number of deaths from all neurological disorders combined was estimated to be 9 million in 2016, representing an increase of 39% between 1990 and 2016 according to the Global Burden of Diseases, Injuries, and Risk Factors Study (GBD) (GBDNeurologyCollaborators, 2019). The rising prevalence of major disabling neurological disorders has been accompanied by increased demand for treatment and medical costs estimated to be nearly $300 billion (GBDNeurologyCollaborators, 2019), exacerbated mainly due to the COVID-19 pandemic (Alzheimer's Association, 2022).

A key shared feature among neurodegenerative diseases is their progressive and incurable nature. Prescribed drugs for neurodegenerative diseases provide symptomatic treatment, but disease-modifying therapeutics are lacking. Moreover, many potential pharmacological therapies have been proven futile in clinical trials. Neurodegenerative diseases, namely Alzheimer's (AD), Parkinson's (PD), Huntington's disease (HD), amyotrophic lateral sclerosis (ALS), frontotemporal dementia (FTD), and multiple sclerosis (MS), are debilitating and chronic diseases affecting older adults and represent a significant threat to human health (GBDNeurologyCollaborators, 2019; Yang et al., 2020).

The etiology of neurodegenerative diseases is highly complex; genetic, environmental factors, and comorbidities influence disease pathogenesis and treatments. It has become evident that a single drug is unlikely to confer neuroprotection. Despite numerous research studies investigating potential biomarkers, the lack of robust diagnostic and prognostic biomarkers for identifying early-stage patients is among the most significant hurdles in clinical trials. In the absence of effective therapeutics, research into lifestyle modifications, including physical activity and diet, for example, has sparked interest among scientists and clinicians.

WHO defines physical activity as any bodily movement of skeletal muscles resulting in energy expenditure (https://www.who.int/news-room/fact-sheets/detail/physical-activity). Physical activity thus encompasses all actions during leisure time and planned exercise in various forms and modalities ranging from low, moderate, to vigorous intensities. In other words, physical activities may include walking, running, and cycling, for example, at any given intensity and frequency. The terms physical activity and exercise are used interchangeably, but some noteworthy differences exist. Physical activity is an umbrella term that covers a wide range of activities conducted in a relatively unstructured manner but includes specific, planned, and structured forms of activities collectively known as physical exercise (Herold et al., 2019).

**Figure 1:**
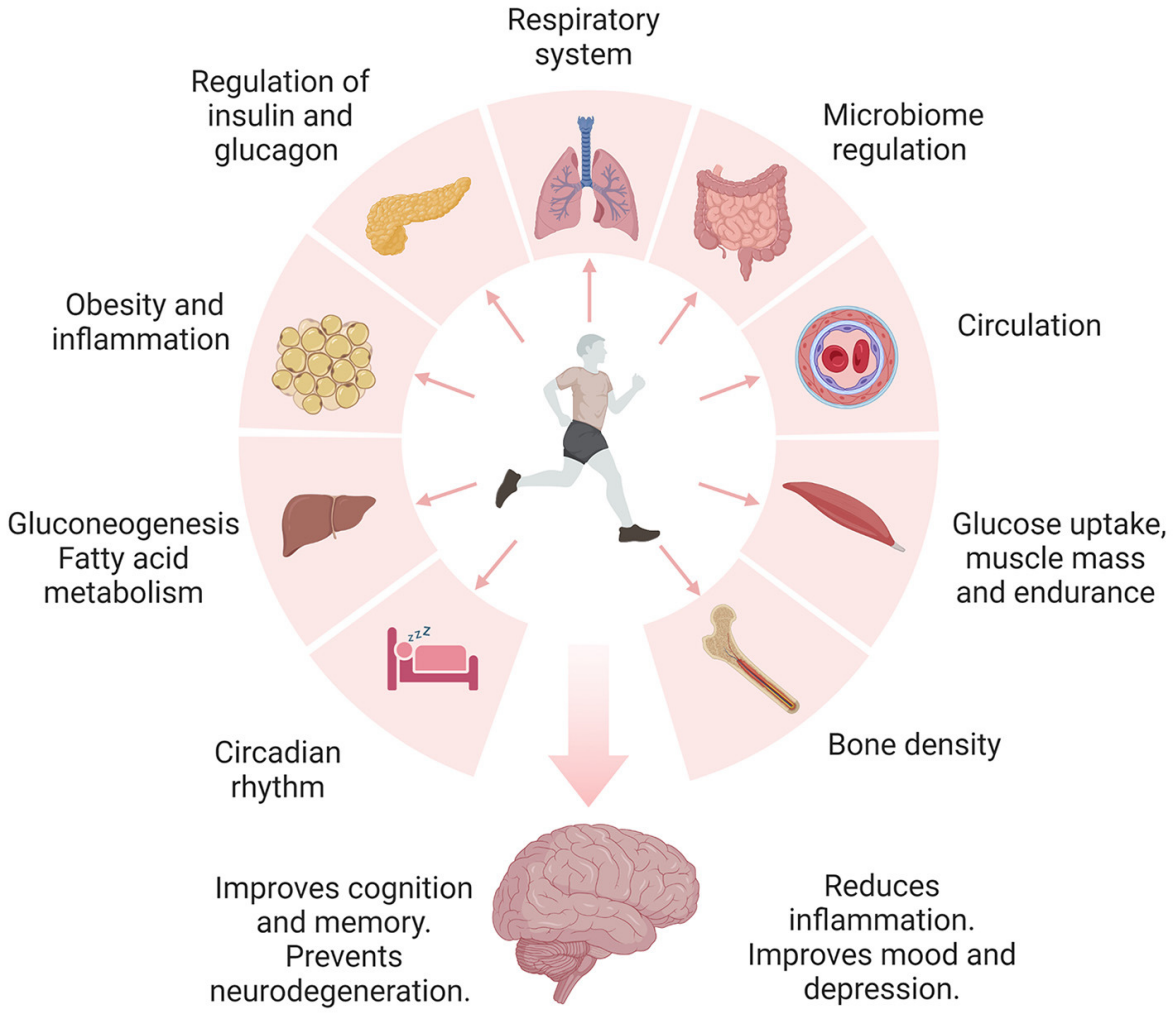
Engaging in daily physical activity and exercise has been shown to promote brain health through the regulation of multiple organ systems. Physical activity improves the regulation of metabolic pathways, including insulin signaling, glucose, carbohydrates, and fatty acid metabolism. Proper control of these pathways is linked to reduced inflammation, insulin resistance, obesity, and diabetes. Additionally, physical activity and exercise improve blood circulation, blood pressure, and respiration. Notably, physical activity and exercise regulate the microbiome and metabolism of essential nutrients. Furthermore, exercising regularly improves bone mass and may prevent osteoporosis. Exercise-induced benefits of sleep include changes in the core body temperature, reduced inflammation, neurotransmitter release, melatonin regulation, and increased expression of growth hormone and BDNF. The release of endorphins during exercise is known to improve mood and depression. Collectively, lifestyle modifications may independently or synergistically guard the brain against neurodegeneration.

It is well-documented that regular physical activity confers numerous beneficial effects across multiple domains, including the cardiovascular, immune, digestive, and central nervous systems (Ruegsegger and Booth, 2018; Figure 1). In the context of brain health, physical activity has been shown to reduce stress, anxiety, and depression and counteract the effects of aging by improving memory and cognitive abilities (Alanko et al., 2022; Ross et al., 2023). Nevertheless, the specific molecular determinants and neuroprotective mechanisms afforded by physical activity still need to be fully characterized. Here, we discuss the studies exploring the effects of physical activity on brain health from several lines of evidence, including but not limited to epidemiological, animal, molecular, and bioinformatic analyses. We provide evidence supporting a multidimensional approach involving physical activity, diet, cognitive training, and sleep for preventing and treating neurodegenerative diseases.

## Physical activity and exercise in Alzheimer's disease dementia

Alzheimer's disease (AD) is a degenerative brain disease and the most common cause of dementia (Arvanitakis et al., 2019). The terms “Alzheimer's disease,” “Alzheimer's disease dementia,” and “dementia” are frequently used interchangeably; however, dementia is not a single disease but rather an umbrella term that covers the characteristic symptoms, which include difficulties with memory, language, problem-solving skills, and other executive functions (Arvanitakis et al., 2019). Misfolded extracellular amyloid β aggregates and intraneuronal neurofibrillary tangles are key disease features (Knopman et al., 2021). According to the Alzheimer's Disease Association, the number of people aged 65 and older with AD dementia is currently 6.5 million and it is expected to reach 13.8 million by 2060, and roughly 10–15% of people with mild cognitive impairment will develop overt dementia each year (Alzheimer's Association, 2022).

As of January 2022, 143 potential drugs were tested in 172 clinical trials for AD (Cummings et al., 2022). However, these medications would only help manage symptoms in people with mild and moderate AD. Numerous clinical trials in AD have proven futile, and many are still ongoing. A recent phase 3 double-blind clinical trial showed moderate clinical benefits of lecanemab, a humanized monoclonal antibody that binds to Aβ soluble fibrils, in early-stage AD (van Dyck et al., 2023). Patients receiving lecanemab displayed a greater reduction in amyloid burden and less cognitive decline than placebo. Notably, adverse events, including infusion reactions and edema, were reported in 26.4% and 12% of participants, respectively. Although promising, extended trials are warranted to evaluate the safety and efficacy of lecanemab in AD.

Given the absence of a modifying treatment for AD, investigations on lifestyle factors, including diet, physical activity, and exercise, have become an appealing alternative for therapeutic development. Different levels of physical activity have been suggested to slow and prevent cognitive decline and progression to dementia. Growing evidence from epidemiological studies suggests that different modalities of physical activity, including light and moderate exercise, can slow cognitive decline and improve behavioral problems in mild cognitive impairment (MCI) and AD patients (Buchman et al., 2012; Table 1). Interestingly, even light physical activity like walking is protective against cognitive decline in older adults (Stubbs et al., 2017). Supporting these findings, a meta-analysis of 15 prospective studies determined that all physical activity levels were protective against cognitive decline in non-demented adults (Sofi et al., 2011). These results are supported by recent studies that have reported the benefits of physical activity against cognitive decline and dementia in several populations. For instance, a prospective cohort in Japan found that a higher level of moderate to vigorous physical activity was associated with a decreased risk of dementia in men (Ihira et al., 2022). Similarly, an increase in physical activity, including light-intensity physical activity, was associated with a reduction in the risk of dementia in Korea (Yoon et al., 2021). In addition, a prospective cohort study in the United Kingdom suggested that a dose of just under 10,000 steps daily was associated with a lower risk of dementia onset (Del Pozo Cruz et al., 2022).

**Table 1:** Epidemiological and clinical studies of physical activity and exercise in mild cognitive impairment and Alzheimer's disease dementia.

**Study type, country, year**	**Sample size**	**Follow up, years**	**Physical activity category, intensity**	**Statistics**	**Main outcome**
Prospective, USA, 2012 (Buchman et al., 2012)	Non-demented: 716	4 yrs	TPA	HR = 0.48 [0.273, 0.832]	A higher level of TPA is associated with a reduced risk of AD.
Prospective, Taiwan, 2017 (Stubbs et al., 2017)	Non-demented: 274	1.84 yrs	LPA	RR = 0.75 [0.60, 0.92]	LPA is associated with reduced cognitive decline.
Meta-analysis, multiple countries, 2011 (Sofi et al., 2011)	15 prospective studies, non-demented: 33,816	1–12 yrs	HPA, LMPA	HPA (HR = 0.62 [0.54, 0.70]) LMPA (HR = 0.65 [0.57, 0.75])	High PA and low to moderate PA were associated with reduced cognitive decline.
Prospective, Japan, 2022 (Ihira et al., 2022)	Non-demented: 43,896	10 yrs	TPA, leisure-time MVPA	TPA (men, HR = 0.75 [0.66, 0.85], women, 0.75 [0.67, 0.84]) and leisure-time MVPA (men, HR = 0.74 [0.65, 0.84] women, 0.74 [0.66–0.83])	TPA and leisure-time MVPA were associated with reduced risk of dementia.
Prospective, Korea, 2021 (Yoon et al., 2021)	Non-demented: 62,286	3.5 yrs	1–499 MET-min/wk Active: 500–999 MET-min/wk Highly active: >1,000 MET-min/wk	Insufficiently active (HR = 0.90 [0.81–0.99]) Active (HR = 0.80 [0.71–0.92]) Highly active (HR = 0.72 [0.60–0.83])	Increased PA was associated with a reduced risk of dementia.
Prospective, United Kingdom, 2022 (Del Pozo Cruz et al., 2022)	Non-demented: 78,328	6.9 yrs	Daily step count	Optimal dose: 9,826 steps (HR = 0.49 [0.39–0.62]) Minimal dose: 3,826 steps (HR = 0.75 [0.67–0.83])	Approximately 10k steps may be optimally associated with a lower risk of dementia.
Meta-analysis, multiple countries, 2016 (Groot et al., 2016)	18 RCTs, dementia: 802	N. A	Aerobic and non-aerobic exercise	HR = 0.41 [0.05–0.76]	Aerobic exercise had a positive effect on cognition in patients with dementia.
Meta-analysis, multiple countries, 2020 (Law et al., 2020)	46 RCTs, MCI and dementia: 5099	N. A	Aerobic exercise	HR = 0.44 [0.27–0.61]	Aerobic exercise reduced cognitive decline in MCI or dementia patients.
Meta-analysis, multiple countries, 2020 (Zhu et al., 2020)	MCI: 842	N. A	Aerobic exercise	HR = 1.43 [0.59–2.27]	Aerobic exercise improves global cognition and executive functions in MCI patients.
Meta-analysis, multiple countries, 2020 (Demurtas et al., 2020)	MCI and dementia: 28,205	N. A	Mixed physical activity	MCI (SMD = 0.30 [0.11–0.49]) Dementia (SMD = 1.10 [0.65–1.64])	PA improved global cognition in MCI and dementia.
Meta-analysis, multiple countries, 2022 (Lopez-Ortiz et al., 2022)	21 studies, >150,000	N. A	Physical activity	AD (OR = 0.60 [0.51–0.71]) Global cognition (SMD = 0.41 [0.24–0.58])	Meeting the WHO recommendations for PA associated with a lower risk of AD and improved global cognition in AD patients.

PA, physical activity; LPA, light physical activity; TPA, total physical activity; LMPA, low to moderate physical activity; MVPA, moderate to vigorous physical activity; OR, odds ratio; HR, hazard ratio; SMD, standardized mean difference; MET, metabolic equivalent of task; RR, risk ratio; N.A, not applicable.

In contrast, mixed results and inconclusive evidence have been reported by other investigations (Brasure et al., 2018; Du et al., 2018). For instance, a meta-analysis of 19 prospective studies showed no significant association between dementia and physical activity when assessing physical activity ≥10 years before dementia onset (Kivimaki et al., 2019). Similarly, other clinical studies have not found a significant association between physical activities and cognitive function, and the risk of AD (Young et al., 2015; Baumeister et al., 2020). Evidence for short-term and single-component physical activity interventions in the prevention of cognitive impairment and dementia is largely insufficient, indicating that multidomain interventions are necessary (Brasure et al., 2018). Reverse causation is a possible explanation for the negative findings. In other words, older adults might stop engaging in physical activities due to symptoms either in the prodromal phase or later in the disease course. The mixed evidence calls for larger randomized multicenter clinical trials assessing different modalities and multidomain interventions in older adults at risk for AD.

## Physical activity and exercise in Parkinson's disease

Parkinson's disease (PD) is the second most common neurodegenerative disease affecting over 8.5 million people globally (https://www.who.int/). Accumulation of misfolded α-synuclein aggregates into intraneuronal inclusions named Lewy bodies is a characteristic pathological feature of the disease. Clinically, PD is characterized by prominent motor symptoms, resting tremors, rigidity, postural instability, and bradykinesia (Poewe et al., 2017). Multiple non-motor symptoms, including cognitive decline, dementia, constipation, hyposmia, restless leg syndrome, and sleep behavior disorder, have been frequently reported in clinical studies (Santiago et al., 2017). Current treatments using carbidopa/levodopa formulations improve motor symptoms, but a disease-modifying drug is yet to be discovered. The search for diagnostic biomarkers for identifying patients at earlier stages of the disease is a pressing matter in the field (Chen-Plotkin et al., 2018).

Cognitive impairment is common in PD patients. Several studies have explored the effects of physical activity on cognition, motor symptoms, and the risk of PD (Table 2). Clinical trials have demonstrated that physical activity is safe, tolerable, and effective in PD patients. For example, a phase 2 randomized trial showed that high-intensity but not moderate treadmill exercise reached the non-futility threshold and decreased motor symptoms in *de novo* PD patients (Schenkman et al., 2018). Phase 3 of this trial is currently underway and will determine the efficacy of high-intensity treadmill exercise in delaying the progression of PD. Recently, moderate-intensity physical activity following the WHO recommendations, but not light physical activity, was associated with improved global cognition, visuospatial function, memory, and executive function in mild-moderate PD patients (Donahue et al., 2022).

**Table 2:** Epidemiological and clinical studies of physical activity and exercise in PD.

**Study type, country, year**	**Sample size**	**Follow up period**	**Physical activity category, intensity**	**Statistics**	**Main outcome**
Meta-analysis, USA, Finland, Sweden, 2018 (Fang et al., 2018)	544,336 (PD: 2,192)	12 yrs	TPA, LPA MVPA	TPA (RR = 0.79 [0.68–0.91]) MVPA (RR = 0.71 [0.58–0.87]), LPA (RR = 0.86 [0.60–1.23])	TPA and MVPA but not LPA were associated with a reduced risk of PD with stronger associations in men.
Meta-analysis, multiple countries, 2022 (Zhen et al., 2022)	PD: 802	N.A	Aerobic exercise	Balance (SMD = 0.99 [0.76–1.23]), gait (SMD = 0.49 [0.20–0.78]), motor function UPDRS-III (SMD = −0.40 [−0.55 to (−0.24)])	Aerobic exercise improved balance (gait and motor function in PD patients.
Cross-sectional, USA, 2018, (Loprinzi et al., 2018)	PD: 25	2 weeks	MVPA	MVPA (MoCA, β = 0.09 [−0.003 to 0.19])	MVPA improved cognition in PD patients.
RCT, phase II, USA, 2022, (Schenkman et al., 2018)	PD: 128	6 months	Treadmill exercise: high and moderate intensity	High-intensity treadmill (UPDRS, 0.3 [−1.7 to 2.3])	High-intensity treadmill but not moderate reached the non-futility threshold.
RCT, phase I/II, USA, 2014, (Uc et al., 2014)	PD: 60	6 months	Aerobic walking	UPDRS-I: Pre (2.1 ± 1.9) Post (1.6 ± 1.3) UPDRS-III: Pre (18.8 ± 10.4) Post (15.9 ± 8.4)	Continuous aerobic exercise improves motor function in mild to moderate PD patients.
Cross-sectional, USA, 2022 (Donahue et al., 2022)	PD: 96	1 week	LPA, MVPA	MVPA, MoCA: β = 0.40 [0.003–0.076] Global cognition: β = 0.015 [0.005–0.025] Executive function: β = 0.017 [0.003–0.030] Memory: β = 0.014 [0.002–0.026] Visuospatial function: β = 0.02 [0.006–0.034]	MVPA but not LPA improved cognition, executive function, and memory in mild-moderate PD patients.
Population-based cohort, USA, 2022 (Zhang et al., 2022b)	PD: 1251	32–24 yrs	Daily physical activity (MET)	HR = 0.35 [0.23–0.52]	Daily physical activity and a healthy diet are associated with a lower rate of all-cause mortality.
RCT, Netherlands, 2019, (van der Kolk et al., 2019)	PD: 130	6 months	Aerobic exercise or stretching	Aerobic exercise: UPDRS 4.2 [1.6–6.9]	Aerobic exercise attenuated motor symptoms assessed by UPDRS.
Open-label, pilot study, Israel, 2007 (Herman et al., 2007)	PD: 9	6 weeks	Intensive treadmill	Pre (UPDRS = 29), gait 1.11 m/s) Post (UPDRS = 22, gait 1.26 m/s)	Intensive treadmill training improved motor symptoms assessed by UPDRS and gait speed.
RCT, China, 2022 (Li et al., 2022)	PD: 95	1 yr	Tai Chi, brisk walking	Tai Chi: Berg balance score (*p* = 0.022), UPDRS total (*p* = 0.015) UPDRS-III (*p* = 0.001)	PD patients in the Tai Chi group had better improvements in balance and motor symptoms.

PA, physical activity, PD, Parkinson's disease, LPA, light physical activity, TPA, total physical activity, LMPA, low to moderate physical activity, MVPA, moderate to vigorous physical activity, OR, odds ratio, HR, hazard ratio, SMD, standardized mean difference, MET, metabolic equivalent of task, MoCA, Montreal Cognitive Assessment, UPDRS, Unified Parkinson's Disease Rating Scale, RCT, randomized controlled trial, RR, risk ratio, N.A, not applicable.

Long-term adherence to exercise routines can be challenging for patients with chronic conditions like PD and AD. A randomized trial employing a motivational mobile application for remote supervision determined that aerobic exercise at home can effectively alleviate some motor symptoms in PD patients with mild disease severity (van der Kolk et al., 2019). In this context, wearable devices can provide real-time monitoring of movements, tremors, and physical activity patterns that may be useful in the clinical management of PD patients (Rovini et al., 2017). Furthermore, wearable technologies are being developed to suppress motor symptoms in PD patients, including hand tremors (Faizan and Muzammil, 2020).

Gait disturbances and falls are common among PD patients, and these may limit the ability to perform physical activities. A small pilot study suggested that intensive treadmill training for 6 weeks improved motor symptoms and enhanced gait rhythmicity in PD (Herman et al., 2007). The therapeutic effect afforded by treadmill training may be facilitated *via* modifications in cerebellum activity (Maidan et al., 2017), reduced pre-frontal cortex activation (Thumm et al., 2018), and changes in interregional connectivity between cortical and subcortical brain regions (Ding et al., 2022).

Tai Chi, sometimes described as “meditation in motion,” is a low-impact and slow-motion exercise with breathing control that has been shown to improve gait and balance in PD. Tai Chi outperformed brisk walking in improving balance, gait, and motor symptoms in PD patients (Li et al., 2022). The improvement of motor symptoms correlated with reduced interleukin 1 beta (IL-1β), L-malic, and phosphoglyceric acids. Additionally, arginine biosynthesis, urea cycle, tricarboxylic acid cycle, and β oxidation of very long chain fatty acids were also improved by Tai Chi. These studies suggest that moderate-intensity physical activity including aerobic exercise, treadmill training, and low-impact activities like Tai Chi can improve motor and non-motor symptoms in PD.

## Physical activity and exercise in rare neurodegenerative diseases

The effects of physical activity have been primarily investigated in aging, AD, and PD studies. Still, its actions on other neurodegenerative diseases, such as ALS, FTD, and HD, have been largely neglected. Contrary to AD and PD, prescribing physical activity interventions in rare neurodegenerative diseases can be challenging due to the disabling nature of these conditions. For example, the progressive degeneration of motor functions and respiratory muscles in ALS significantly limits the ability to perform physical activities. Nevertheless, several studies have shown that various modalities of physical activity can be safe and may be effective in improving the quality of life of ALS patients (Clawson et al., 2018) and other rare neurodegenerative diseases (Table 3). For instance, a small study showed that resistance exercise improved the functional capacity of the upper and lower extremities in ALS patients with no adverse events (Bello-Haas et al., 2007). The combination of aerobic and resistance training improved or maintained the physical function of ALS patients (Ferri et al., 2019; Zhu et al., 2022), albeit without improvement in muscle function. Recently, the use of motor-assisted movement exercisers (MME) for a minimum of five sessions per week had a beneficial effect on preserving and improving muscle strength and general wellbeing in ALS (Maier et al., 2022).

**Table 3:** Epidemiological and clinical studies of physical activity and exercise in rare neurodegenerative diseases.

**Study type, country, year**	**Sample size, disease**	**Follow up period**	**Physical activity category, intensity**	**Statistics**	**Main outcome**
RCT, USA, 2018 (Clawson et al., 2018)	ALS: 59	12 and 24 weeks	Resistance, endurance, stretching/range of motion	N.A	All three forms of exercise are safe and tolerated.
RCT, Canada, 2007 (Bello-Haas et al., 2007)	ALS: 33	6 months	Resistance	Total ALSFRS score (*t* = −2.05, *df* = 23, *p* = 0.05)	Resistance training improved ALSFRS functional scores.
Meta-analysis, multiple countries, 2022 (Zhu et al., 2022)	10 RCT studies, ALS	N.A	Aerobic, resistance, standard rehabilitation, passive exercise, daily activity	Probability rank: quality of life (probability = 0.64), fatigue (probability = 0.39), physical function (probability = 0.51)	Combined aerobic and resistance exercises and traditional rehabilitation reduced fatigue and improved quality of life.
Cross-sectional, Germany, 2022 (Maier et al., 2022)	ALS: 144	N.A	Motor-assisted movement exerciser	Reduction of muscle stiffness (*p* = 0.011), limbs rigidity (*p* = 0.0280, improved general wellbeing (*p* = 0.048)	MME improved rigidity, muscle stiffness, and general wellbeing.
RCT, Italy, 2019 (Ferri et al., 2019)	ALS: 16	12 weeks	Aerobic and strength	The gas exchange threshold increased from 0.94 ± 0.08 to 1.06 ± 0.10 (*p* = 0.009, ES = 0.47)	Combined aerobic and strength training 60 min 3 times per week improved aerobic fitness and maintained physical function.
Open-label, Denmark, 2017 (Jensen et al., 2017)	ALS: 12	12 weeks	Resistance	ALSFRS-R scores Baseline: 40.2 ± 2.3 Pre-exercise: 38.6 ± 1.9 Post-exercise: 35.2 ± 4.3	Resistance training did not improve physical function.
RCT, Italy, 2019 (Zucchi et al., 2019)	ALS: 65	2 yr	High intensity (Aerobic, endurance, stretching)	ALFRS-R scores 3 months: 34.87 ± 8.49, *p* = 0.48 12 months: 30.16 ± 9.78, *p* = 0.72 24 months:27.25 ± 9.20, *p* = 0.73	High-intensity physical exercise did not improve ALSFRS scores, motor, and respiratory functions, fatigue, and survival.
Population-based, case-control, Netherlands, 2013 (Huisman et al., 2013)	ALS: 636	N.A	Leisure time PA	OR = 1.08 [1.02,1.14]	Leisure time PA associated with an increased risk of ALS.
Prospective cohort, USA, 2020 (Casaletto et al., 2020)	105 mutation carriers (C9orf72, MAPT, GRN) 69 non-carriers	3 yrs	Leisure time PA	PASE × time β = −0.11, *p* = 0.016 CAS × time β = −0.13, *p* = 0.003	Greater physical and cognitive activities were associated with less functional decline and better cognitive performance.
Prospective cohort, USA, 2022 (Casaletto et al., 2023)	160 mutation carriers (C9orf72, MAPT, GRN)	4 yrs	Leisure time PA	Plasma Nfl (β = −0.13; *b* = −0.002, SE = 0.001, *p* = 0.03)	Higher PA was associated with lower levels of plasma Nfl, a marker indicative of axonal damage.

ALSFRS, ALS functional rating scale; CAS, Cognitive Activity Scale; df, degrees of freedom; ES, effect size; GRN, progranulin; PA, physical activity; LPA, light physical activity; TPA, total physical activity; LMPA, low to moderate physical activity; MAPT, microtubule-associated protein tau; MVPA, moderate to vigorous physical activity; OR, odds ratio; PASE, Physical Activity Scale for the Elderly RCT, randomized controlled trial; t, *t*-test; N.A, not applicable.

In contrast, several studies have shown no benefit from physical activity and that it may be a risk factor for ALS. For example, a resistance training program did not improve neuromuscular function in ALS patients (Jensen et al., 2017). Similarly, an exercise regime consisting of aerobic exercise, endurance, and stretching did not improve motor and respiratory functions, and the quality of life of ALS patients (Zucchi et al., 2019).

Evidence from larger studies has suggested that physical activity may increase the risk of ALS. For instance, a higher level of leisure-time physical activity was associated with an increased risk of ALS, and no differences were found in the level of physical activities, including marathons and triathlons (Huisman et al., 2013). A multicenter study of three European countries determined a positive association between physical activity and the risk of ALS (Visser et al., 2018). Recently, a Mendelian randomization study observed that moderate to vigorous physical activity could increase the risk of ALS in individuals of European ancestry (Liao et al., 2022). Several studies have reported a higher risk of ALS among athletes, particularly professional soccer, and football players (Chio et al., 2005; Lehman et al., 2012).

Several hypotheses to explain these associations have been posited. Concussions and traumatic brain injuries are common among athletes, and it has been suggested that repetitive head injuries contribute to lower resilience to neurodegeneration and increased risk of ALS (Chen et al., 2007), AD-like dementias (Ramos-Cejudo et al., 2018), and PD (Delic et al., 2020). Nevertheless, this association remains debated (Armon and Nelson, 2012). Indeed, a history of head injury did not correlate with disease progression or neuropathological changes in ALS (Fournier et al., 2015).

Frontotemporal dementia (FTD) shares remarkably similar pathogenic mechanisms with ALS. FTD is characterized by the progressive degeneration of the frontal and temporal lobes of the brain. Approximately 50% of FTD cases are familial and associated with mutations in microtubule-associated protein tau (MAPT), progranulin (GRN), and C9orF72 (Wood et al., 2013). The first study showed that greater physical and cognitive activities were associated with an estimated 55% less functional decline in autosomal dominant FTD (Casaletto et al., 2020). Strikingly, autosomal dominant FTD-mutation carriers in C9orF72, MAPT, and GRN, who engaged in physical and cognitive activities, showed improved functional and cognitive trajectories despite their brain atrophy compared to their less active peers. This study suggested that physical activity confers neuroprotection through mechanisms that can influence brain structure functions, such as inflammation and synaptic signaling, rather than directly altering brain structure (Casaletto et al., 2020). A recent follow-up study by the same group showed that FTD subjects carrying autosomal dominant variants who engaged in higher physical activity displayed lower levels of neurofilament light chain (NfL), a marker of axonal degeneration (Casaletto et al., 2023).

Huntington's disease (HD) is a rare autosomal dominant neurodegenerative disease with an estimated prevalence of 2.71 per 100,000 people (Pringsheim et al., 2012). The mean age of onset is 45 years, and some characteristic symptoms include involuntary movements, memory loss, and personality changes (Tucci et al., 2022). Several clinical trials have shown that physical activity is safe, feasible, and effective in improving motor and cognitive function in HD patients. A small randomized trial showed that physical activity improved the cognitive scores assessed by the SF-36 Mental Component Summary but did not improve the Unified Huntington Disease Rating Scale cognitive scores (Busse et al., 2013). Another trial indicated that aerobic and resistance exercises reduced motor symptoms, including chorea and postural instability (Thompson et al., 2013). These studies demonstrate that engaging in physical activity has the potential to modify disease even in autosomal dominant disorders.

## Physical activity in multiple sclerosis

Multiple sclerosis (MS) is an immune-mediated neurodegenerative disease characterized by the degeneration of myelin sheaths by auto-reactive lymphocytes. MS usually presents with earlier onset than other neurodegenerative diseases. The worldwide prevalence of MS is estimated to be approximately 2.8 million people, with an increased prevalence in women (Walton et al., 2020). Strikingly, in the United States, nearly one million people live with MS, and 74% are female patients (https://www.nationalmssociety.org/). Drugs prescribed to MS patients work by downregulating the immune response and have shown beneficial effects during the early stages of the disease (Torkildsen et al., 2016). Therapeutics targeting the immune system have gained interest since a recent study found a link between the Epstein-Barr virus and MS (Lanz et al., 2022).

Decades ago, clinicians did not recommend exercise to MS patients because it would increase fatigue and worsen symptoms (Proschinger et al., 2022). This assumption, however, was proven erroneous when physical activity and exercise programs were shown to be safe and effective in improving symptoms, restoring functions, and optimizing the overall quality of life of MS patients (Motl et al., 2017). Several clinical studies have indicated that physical activity and exercise benefit patients with MS. For example, aerobic exercise with low to moderate intensity, stretching, and flexibility exercises have successfully improved fatigue and reduced muscle spasticity and painful contractions in patients with mild or moderate MS (Halabchi et al., 2017). Indeed, clinical studies have demonstrated improvement in clinically relevant scales used to assess disability in MS patients. For instance, cross-sectional studies have shown a negative correlation between physical activity and the Expanded Disability Status Scale (EDSS), a reliable indicator of disability in MS (Table 4). Furthermore, some studies indicate that physical activity is associated with gray and white matter brain volumes in brain structures involved in motor and cognitive functions (Klaren et al., 2015; Kalron et al., 2020). Recently, moderate to vigorous physical activity was positively associated with axonal and neuronal integrity in MS patients (Kim et al., 2022). These studies suggest that different physical activity levels improve symptoms and maintain and preserve brain structures important for motor and cognitive abilities in MS patients. The epidemiological and clinical studies addressing the impact of physical activity and exercise in MS patients have been described in detail elsewhere (Proschinger et al., 2022).

**Table 4:** Epidemiological and clinical studies of physical activity and exercise in multiple sclerosis.

**Study type, country, year**	**Sample size**	**Follow-up, time**	**Physical activity category, intensity**	**Statistics**	**Main outcome**
Cross-sectional, USA, 2011 (Cavanaugh et al., 2011)	MS:21	1 week	Daily step count	*r* = −0.90, *p* < 0.01	Total step counts count correlated negatively with EDSS.
Cross-sectional, USA, 2015 (Fjeldstad et al., 2015)	MS: 13 HC: 12	1 week	Daily step count (accelerometer)	*r* = −0.61, *p* < 0.05	Total weekly step counts correlated negatively with EDSS.
Cross-sectional, USA, 2015 (Klaren et al., 2015)	MS: 39	1 week	PA level (accelerometer) Sedentary ( ≤ 100 counts/min) LPA (100–1,722 counts/min) MVPA (≥1,723 counts/min)	Hippocampus (*pr* = 0.49, *p* < 0.01) Thalamus (*pr* = 0.38, *p* < 0.05) Caudate (*pr* = 0.54, *p* < 0.01) Putamen (*pr* = 0.37, *p* < 0.05) Pallidum (*pr* = 0.50, *p* < 0.01)	MVPA was associated with whole brain gray and white matter volumes and brain structures involved in motor and cognition in MS patients.
Cross-sectional, 2017, USA (Block et al., 2017)	MS: 99	>4 weeks	Daily step count (accelerometer)	*r* = −0.71, *p* < 0.001	Daily step count correlated negatively with EDS. Lower PA was associated with greater disability.
Prospective, 2019, USA (Block et al., 2019)	MS: 95	1 year	Daily step count (accelerometer)	OR = 4.01, 95% CI [1.17–13.78], *p* = 0.03	Participants with an average daily step count below 4,766 had higher odds of disability according to the EDSS score.
Cross-sectional, retrospective, 2020, Israel (Kalron et al., 2020)	MS: 153	N.A	Leisure time PA	Physically active: hippocampus (48.5, S.D = 32.2) Insufficiently active: hippocampus (34.6, S.D = 30.8, *p* = 0.004)	Patients who engaged in regular PA maintain their hippocampal volume.
RCT, 2004, Finland (Romberg et al., 2004)	MS: 95	6 months	Strength and aerobic training	Physically active: the 7.62 m walk test time decreased by 12% (95% CI 15% to 7%, *p* < 0.001).	Exercising patients improved their walking speed assessed with the 7.62m and 500 m walking tests.
RCT, 2015, Belgium (Wens et al., 2015)	MS: 34	12 weeks	High-intensity exercise High-intensity cardiovascular training	Mean muscle fibers crossectional area (HIT:+21 ± 7%, HCT:+23 ± 7%)	High-intensity interval (HIT) and continuous cardiovascular exercise (HCT) was safe and increased mean muscle fibers‘crossectional area.
Cross-sectional, 2022, USA (Kim et al., 2022)	MS: 41 HC: 79	1 week	MVPA	RFNL (*r* = 0.38, *p* < 0.01) TMV (*r* = 0.49, *p* < 0.01)	MVPA correlated with retinal nerve fiber thickness (RNFL) and total macular volume (TMV).

EDSS, Expanded Disability Status Scale PA, physical activity; LPA, light physical activity; MVPA, moderate to vigorous physical activity; OR, odds ratio; pr, partial correlation; RCT, randomized controlled trial; N.A, not applicable.

In addition to physical activity and exercise, nutrition is an important determinant of health outcomes in MS patients and other neurodegenerative diseases. Several dietary approaches have shown promise in improving symptoms in MS patients. Low saturated fat, low fat vegan, modified Paleolithic (Wahls), gluten-free, Mediterranean, intermittent fasting, and calorie restriction have been investigated, showing promising results in alleviating symptoms in MS patients (Chenard et al., 2019; Wahls, 2022). Some of these dietary approaches, including the modified Paleolithic and Mediterranean diets, have been associated with significant clinical improvement (Lee et al., 2021; Katz Sand et al., 2023). Notably, these dietary approaches have proven useful in modifying comorbidities, including diabetes, cardiovascular disease, and depression, which are largely implicated in neurodegenerative diseases. Larger longitudinal trials evaluating these dietary interventions are warranted.

## Physical activity modifies risk factors associated with neurodegenerative diseases

A sedentary lifestyle is a prime contributor to the development of chronic diseases such as obesity, type II diabetes mellitus (T2DM), and cardiovascular disease. Physical inactivity was declared the fourth cause of death worldwide, according to the WHO (Bull et al., 2020). The 2022 Global status report by the WHO indicates that approximately 500 million people will develop heart disease, obesity, and T2DM resulting from physical inactivity by 2030 (https://www.who.int/publications/i/item/9789240059153).

Epidemiological studies have indicated that physical activity modifies cardiovascular disease, obesity, and T2DM. Earlier prospective cohort studies suggested that greater physical activity was associated with a reduced risk of T2DM in women (Hu et al., 1999), cardiovascular disease, and mortality in adults with T2DM (Gregg et al., 2003; Tanasescu et al., 2003). In addition, randomized controlled trials have indicated that engaging in physical activity is inversely associated with the risk of cardiovascular events in patients with impaired glucose tolerance (Yates et al., 2014). Interestingly, lifestyle intervention alone was more effective at reducing the risk of T2DM than metformin. A 67% reduced risk of T2DM was observed in subjects who received instructions on lifestyle interventions, including physical activity and diet (Kosaka et al., 2005). These findings are important considering the numerous studies that have shown a potential link between T2DM, cardiovascular disease, and neurodegenerative diseases (Santiago and Potashkin, 2013, 2021; Potashkin et al., 2020). Similarly, these results highlight the potential of physical activity and diet as adjunct therapies for disease modification.

T2DM shares several molecular pathways with AD and PD, suggesting a common disease etiology. Mitochondrial dysfunction, endoplasmic reticulum stress, insulin resistance, vascular abnormalities, and inflammation are some shared mechanisms between T2DM, PD, and AD (Santiago and Potashkin, 2013, 2021). In addition, a diagnosis of T2DM has been shown to impact the worsening of symptoms and disease progression in AD and PD (Santiago and Potashkin, 2021; Athauda et al., 2022). The ample evidence from epidemiological and molecular studies has fueled the investigations on commonly prescribed anti-diabetic drugs as potential therapeutics for AD and PD (Reich and Holscher, 2022).

These studies on T2DM and neurodegeneration have underscored the critical importance of studying comorbidities in personalized medicine applications and have paved the way for new therapeutic interventions in neurodegenerative diseases. One plausible neuroprotective mechanism afforded by physical activity is the modification and prevention of T2DM and obesity, frequently associated with an increased risk of neurodegeneration.

## Neuroprotective mechanisms mediated by physical activity: evidence from pre-clinical models and bioinformatic approaches

Pre-clinical studies have reported beneficial effects of exercise in cognitive functions, memory preservation, and the prevention of neurodegenerative diseases. Regular exercise reduced the expression of amyloid-β and phosphorylated tau and increased synaptic activity and expression of glucose transporters GLUT1 and GLUT3 in AD model mice (Pang et al., 2019). Voluntary running exercise increased microglial glucose metabolism and protein expression of GLUT5, triggering receptor expressed on myeloid cells 2 (TREM2), secreted phosphoprotein 1 (SPP1), and phosphorylated spleen tyrosine kinase (p-SYK) in the hippocampus of APP/PS1 mice compared to the sedentary group (Zhang et al., 2022a). Short-term resistance exercise improved cognition, reduced amyloid-β, and hyperphosphorylated tau brain deposits, and inhibited the expression of neuroinflammatory markers tumor necrosis factor alpha (TNF-α) and IL-1β (Liu et al., 2020). Treadmill running lowered amyloid β burden and neuroinflammatory markers and improved mitochondrial function in the hippocampus and cerebral cortex of triple transgenic AD mice (3xTg-AD) (Kim et al., 2019).

Similarly, prolonged voluntary wheel running improved spatial memory performance, increased dendritic spines, and reduced extracellular amyloid β accumulation in 3xTg-AD mice (Xu et al., 2022). Long-term voluntary running reversed cognitive impairment, increased glial fibrillary acidic protein (GFAP) immunoreactivity, and astrocytic brain-derived neurotrophic factor (BDNF) in the hippocampus of 5xFAD mice (Belaya et al., 2020). Treadmill exercise alleviated cognitive decline and β-amyloid neurotoxicity *via* furin-mediated iron regulation (Choi et al., 2021). Physical activity alleviated cognitive impairment and neuroinflammation *via* upregulation of miR-129-5p in APP/PS1 AD mice (Li et al., 2020). Boosting the expression of irisin, an exercise-induced myokine, rescued synaptic plasticity and memory in APP/PS1 AD mice (Lourenco et al., 2019). Infusion of plasma from mice exposed to exercise for 3 months into 3xTg AD mice improved cognitive function and neuroplasticity and suppressed apoptosis (Kim et al., 2020).

Running exercise slowed the decline in spatial learning and memory abilities in male and female APP/PS1 mice. In addition, there was an increase in the myelinated fibers of the white matter in male AD mice compared to females (Zhou et al., 2018). Aerobic exercise decreased cognitive impairment and increased myelination in C57/BL-aged mice through the upregulation of ROCK signaling (Bao et al., 2021), a central pathway in myelination and axon growth in the central nervous system (Fujita and Yamashita, 2014).

In the context of PD, physical exercise improved motor function, reduced cognitive impairment, and modulated the expression of L-DOPA, cAMP-responsive element binding protein 1 (CREB1), and RPTOR independent companion of MTOR complex 2 (RICTOR), genes involved in mitochondrial function and dopamine signaling in 1-methyl-4-phenyl-1,2,3,6-tetrahydropyridine (MPTP)-treated mice (Aguiar et al., 2016; Klemann et al., 2018). Furthermore, it has been suggested that the observed recovery in motor functions afforded by physical activity is facilitated *via* the enhancement of dopamine transporters and the downregulation of the inflammatory response (Churchill et al., 2017). In addition, physical exercise normalized the expression of genes involved in the receptor for advanced glycation products (RAGE), critical effectors of the innate immune response (Viana et al., 2017). Furthermore, physical activity upregulated the neuroprotective factor PD-related DJ-1 in the frontal cortex of MPTP-treated rats (Viana et al., 2017).

Bioinformatic approaches from human gene expression datasets have been instrumental in delineating important biological and molecular mechanisms associated with physical activity in the brain. Differentially expressed genes in the hippocampus of physically active subjects inversely correlated with those from AD and aging individuals (Berchtold et al., 2019). These genes were enriched in mitochondrial energy production and synaptic function. Similarly, high physical activity induced the expression of genes associated with neurogenesis and T-cell-mediated inflammation in the hippocampus of cognitively intact individuals (Sanfilippo et al., 2021). Similarly, our studies showed that physical activity induces dramatic transcriptional changes in the hippocampus of cognitively intact individuals. We found that gene expression patterns induced by physical activity inversely correlated with those from neurodegenerative diseases, including AD, PD, FTD, and HD (Santiago et al., 2022).

Interestingly, physical activity mediated its effects through different pathways across neurodegenerative diseases. For instance, physical activity induced the upregulation of genes involved in synaptic signaling in AD, PD, and HD. In FTD, differentially expressed genes were enriched in bioenergetic processes and the generation of energy precursors (Santiago et al., 2022). Furthermore, physical activity mediated the downregulation of inflammation-related genes in AD.

Metabolomic studies have helped study gut microbiome alterations in neurodegenerative diseases. Low physical activity was associated with fecal metabolome differences in PD patients. Reduced short-chain fatty acids and butyrate levels correlated with cognitive impairment and worse postural instability-gait scores (Tan et al., 2021). Metabolomic analysis revealed that treadmill training increased polyunsaturated fatty acids, cathepsin B (CTSB), and reduced ceramides, sphingolipids, and BDNF levels in the plasma of asymptomatic late middle-aged adults at risk for AD (Gaitan et al., 2021). Increased levels of plasma CTSB correlated positively with cognitive performance. Similarly, treadmill exercise reduced depressive symptoms and increased the Firmicutes/Bacteroidetes ratio, improving gut dysbiosis in mice treated with Aβ_1 − 40_ (Johnston and Barker, 1987). These studies indicate that physical activity helps regulate the microbiome and metabolism of essential nutrients, thereby promoting neuroprotection. Recently, a microbiome-dependent gut–brain axis was found to regulate motivation for exercise and performance. An intact gut microbiome contributes to the generation of intestinal fatty acid amides that trigger cannabinoid receptor 1-expressing neurons to send a signal to the brain that promotes the downregulation of monoamine oxidase, thereby increasing dopamine signaling in the brain and enhancing physical performance and motivation (Dohnalova et al., 2022).

## Physical activity, nutrition, sleep, and mindfulness meditation: a multidimensional approach to brain health

Though engaging in physical activity has been well-established to promote brain health overall, environmental, genetic, and socioeconomic factors also play a fundamental role in human health. Indeed, a recent study indicated that the environment in which exercise is performed plays an equally important role as the exercise itself, suggesting that outdoor physical activity may be better for brain health (Boere et al., 2023).

**Figure 2:**
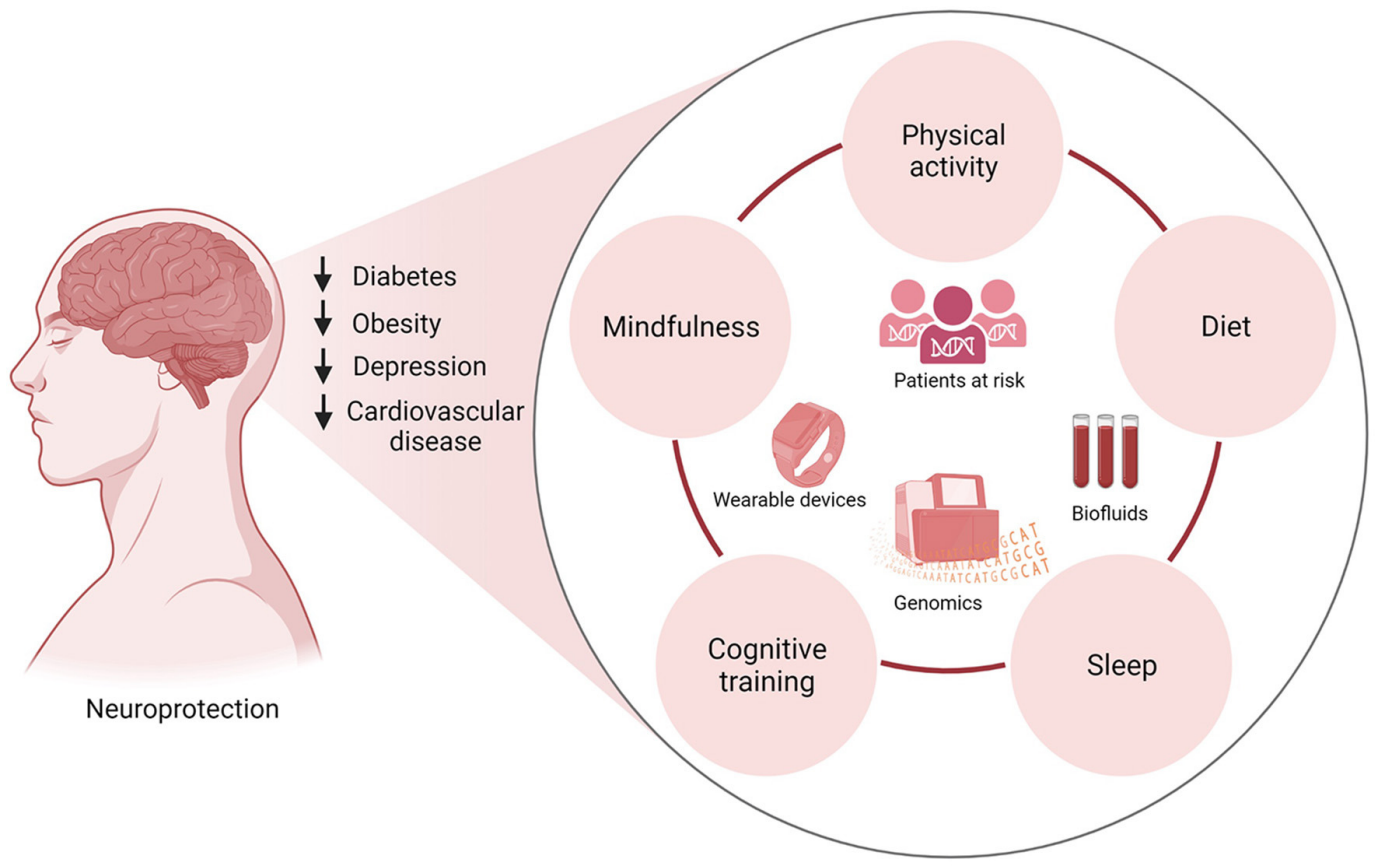
Multidomain clinical trials integrating physical activity, diet, cognitive training, mindfulness, and sleep hygiene in patients at risk of neurodegenerative diseases can inform lifestyle interventions that promote neuroprotection. Lifestyle modifications have been shown to reduce the risk of diabetes, obesity, cardiovascular disease, and depression, common conditions associated with dementia and neurodegenerative diseases. Incorporating lifestyle changes such as physical activity, eating a nutrient-rich diet, restful sleep, cognitive training, and mindfulness will reduce the risk of these chronic diseases. The implementation of wearable technologies to track participants' sleep patterns and physical activity in conjunction with the collection of biofluids for gene expression studies can lead to the identification of biomarkers and potential therapeutics for clinical intervention.

Given the complex etiology of neurodegenerative diseases, multidomain interventions are more likely to delay or prevent disease onset. Emerging studies geared toward personalized medicine have begun investigating the adjunct use of physical activity with other lifestyle interventions (Figure 2). For instance, several studies have investigated the synergy between physical activity, and cognitive training, for the prevention of neurodegenerative diseases. Both cognitive therapy and physical activity improved global cognitive function in patients with MCI and dementia (Wang et al., 2014; Karssemeijer et al., 2017). This is an important finding considering recent investigations that have found that reducing time spent in cognitively passive activities such as watching TV and increasing those that require active cognition may be more effective at reducing the risk of dementia (Raichlen et al., 2022). While physical activity alone can be beneficial, sedentary behaviors can dangerously counteract the benefits of physical activity and trigger neurodegeneration.

Diet is another key element in promoting overall health. Engaging in physical activity and healthy dietary patterns were associated with a lower rate of all causes of mortality in PD patients (Zhang et al., 2022b). In addition, a multidomain intervention showed that following a Mediterranean diet, engaging in physical activity, and cognitive training improved cognitive function in older adults at risk of dementia (Ngandu et al., 2015). Additional multidomain trials to increase Mediterranean diet adherence and physical activity in older adults at risk of dementia are underway. These trials will be crucial to determine if combined lifestyle interventions can prevent dementia (Heffernan et al., 2019; Shannon et al., 2021).

Sleep disorders are common in neurodegenerative diseases and correlate with cognitive and neuropsychiatric problems (Memon et al., 2020). Dysregulation in the sleep–wake cycle has been shown to promote the accumulation of amyloid-β, tau, and synuclein in the interstitial fluid of mice and CSF in humans (Holth et al., 2019). Strikingly, sleep deprivation increased tau by 50% and amyloid- β by 30% in human CSF (Lucey et al., 2018; Holth et al., 2019). Evidence from animal models showed that chronic sleep deprivation increased tau pathology spreading (Holth et al., 2019). A longitudinal study using data from 7959 subjects and a 25-year follow-up determined that sleeping less than 6 h at ages 50, 60, and 70 was associated with a 30% increased dementia risk (Sabia et al., 2021). Another study found that midlife and late-life insomnia were associated with a higher dementia risk (Sindi et al., 2018). The same study determined that sleeping more than 9 h was also associated with an increased risk of dementia.

Several exercise modalities, including aerobic, resistance, and Tai Chi, have improved sleep outcomes in patients with neurodegenerative diseases. Randomized trials have indicated that walking daily for 30 min improved sleep outcomes in patients with AD (McCurry et al., 2005, 2011). Mild to moderate aerobic exercise attenuated sleep disturbances in AD and PD patients (Nascimento et al., 2014).

Several potential mechanisms underlying exercise-induced benefits of sleep include changes in the core body temperature, reduced inflammation, serotonin, norepinephrine, and melatonin regulation, and increased expression of growth hormone and BDNF (Memon et al., 2020). Despite the benefits shown in AD and PD, to the best of our knowledge, there is a lack of studies exploring the effects of physical activity on sleep disturbances in ALS and HD.

In addition to sleep disturbances, stress, depression, and anxiety are commonly observed in older adults and increase the risk of dementia (Wilson et al., 2002). Meditation and mindfulness practices have been shown to reduce these psycho-affective states, improve cognition, and preserve brain structure and function (Gard et al., 2014; Chetelat et al., 2018). For example, a randomized trial showed that mindfulness training prevented depression in early-stage AD patients in a 2-year follow-up (Quintana-Hernandez et al., 2023). Another trial demonstrated that mindfulness meditation improved cognitive performance and connectivity between the hippocampus and posteromedial cortex in healthy adults (Sevinc et al., 2021). Interestingly, neuroimaging studies in expert meditators revealed increases in gray matter volume and glucose metabolism in sensitive regions affected by AD, including the prefrontal, anterior, and posterior cingular cortices, insula, and temporoparietal regions (Chetelat et al., 2017). Long-term meditation is associated with thickening in the prefrontal cortex and right anterior insula (Lazar et al., 2005). In addition, increased telomerase activity in blood leukocytes and reduced expression of inflammatory markers have been reported in experienced meditators (Schutte and Malouff, 2014). Finally, it has been proposed that mindfulness and cognitive training improve cognition through increased BDNF levels (Angelucci et al., 2015; Nicastri et al., 2022). These studies suggest that mindfulness training can reduce psycho-affective risk factors associated with dementia and preserve the function of brain regions implicated in cognition, memory, and emotions. Additional trials incorporating sleep, diet, cognitive therapy, and physical activity will help determine whether multidomain interventions are more effective than single interventions in preventing and treating neurodegenerative diseases.

## Challenges and future directions

Physical activity is the single most accessible lifestyle modification that has been shown to confer protection against many diseases. For example, a recent study indicated that 10,000 steps a day are associated with a lower risk of all-cause mortality, including cancer and cardiovascular disease (Del Pozo Cruz et al., 2022). Protection against neurodegenerative diseases is mediated through a wide range of biological mechanisms, including reduced inflammation, increased synaptic signaling, improved blood circulation, homeostatic control of glucose and cholesterol levels, regulation of the sleep–wake cycle, and the intestinal microbiome. More indirectly, physical activity modifies well-established risk factors and comorbidities associated with neurodegeneration, including T2DM, obesity, cardiovascular disease, and depression.

Following the WHO recommendations of daily physical activity has shown promise in slowing cognitive decline and mortality. However, prescribing physical activity as an adjunct treatment poses several challenges to clinicians. The intensity, frequency, and modality of exercise may vary from person to person and the target disease. For example, walking and treadmill running have shown benefits for AD and PD patients, whereas resistance training has been a better modality in ALS patients. The challenges of prescribing physical activity as an adjunct treatment for neurodegenerative diseases are not any different from those of personalized medicine. The prescription of exercise as an adjuvant therapy should consider factors beyond those that are non-modifiable such as sex and genotype. Interindividual heterogeneity of patients suffering from different neurodegenerative diseases in response to acute, chronic, and different intensities of exercises should be considered (Herold et al., 2019). An exercise regime tailored for each patient according to their neurodegenerative disease phenotype and comorbid conditions would be ideal for improving health outcomes. Similarly, incorporating a diet plan and a sleep hygiene protocol for each patient would be ideal. Finally, implementing wearable technologies for improving diagnosis, tracking the progression of motor symptoms, encouraging increased physical activity, and tracking compliance may help transform many aspects of the clinical management of patients. Multidomain clinical trials investigating the integrative effect of exercise, diet, cognitive therapy, mindfulness, and sleep on individuals at high risk will be crucial in determining the best combination of lifestyle modifications for the prevention and treatment of neurodegenerative diseases.

## Author contributions

JS: writing—original draft preparation. JS and JP: writing—review and editing. JP: funding acquisition. All authors have read and agreed to the published version of the manuscript.
